# 89. The Impact of Nirsevimab on an RSV Season in All Infants: Data From The HARMONIE Study

**DOI:** 10.1093/ofid/ofad500.005

**Published:** 2023-11-27

**Authors:** Saul N Faust, Katrina Cathie, S B Drysdale, S Royal, C Felter, N C Vassilouthis, Mathieu Bangert, K Mari, R Nteene, M Roberts, M Knuf, F Flamein, P Tissieres

**Affiliations:** University of Southampton and University Hospital Southampton NHS Foundation Trust , Southampton, England, United Kingdom; University of Southampton, Southampton, England, United Kingdom; St George's, University of London, London, England, United Kingdom; University of Nottingham, Nottingham, England, United Kingdom; Sanofi, Reading, England, United Kingdom; Sanofi, Reading, England, United Kingdom; Sanofi Vaccines, Lyon, France, Lyon, Rhone-Alpes, France; Sanofi, Reading, England, United Kingdom; Sanofi, Reading, England, United Kingdom; Sanofi, Reading, England, United Kingdom; Klinikum Worms, Worms, Rheinland-Pfalz, Germany; CHU Lille, Lille, Lorraine, France; APHP, Paris, Ile-de-France, France

## Abstract

**Background:**

RSV is an extremely common respiratory pathogen and a leading cause of infant hospitalisation. An estimated 1 in 7 infants will develop an RSV lower respiratory tract infection (LRTI) requiring medical attention. The majority of infants hospitalised have no comorbidities and were born at term. Nirsevimab is the only preventative option designed to provide protection to all infants from RSV LRTI, from the start of their first RSV season, for the duration of that season.

In the HARMONIE trial conducted in the UK, France, and Germany (EudraCT 2022-000099-20), we evaluated the impact of nirsevimab on all cause LRTI hospitalisations, as well as RSV LRTI specifically. Analyses looked at the efficacy of nirsevimab across subgroups that constitute the all infant population, all of whom are vulnerable throughout their first RSV season.

**Methods:**

Individually randomised infants (≥29 weeks gestational age) received a single intramuscular injection of nirsevimab (< 5 kg 50 mg; ≥5 kg 100 mg), or no intervention (standard of care) before or during the RSV season. Following a single physical visit participants were monitored remotely for all cause LRTI hospitalisation (secondary endpoint, defined as treating physician decision to admit to in-patient care for >24 hours). Efficacy was evaluated through the RSV season. Adverse events (AEs) continue to be monitored for 365 days.

**Results:**

8058 infants were randomized, 4037 to the nirsevimab group and 4021 to the no intervention group. Efficacy against RSV LRTI was 83.21% (CI 67.77-92.04%) across all countries. Efficacy was consistent across infant subgroups, in those born at term (≥ 37 weeks: 84.41% (CI 64.92-94.10%)) or prematurely (< 37 weeks: 78.31% (CI 33.49-94.69%)) and not impacted by infant weight at randomisation (< 5kg: 82.12% CI 59.14-93.30% and ≥ 5kg 85.16% CI 57.01-96.25%). Efficacy against all cause LRTI hospitalisation was 58.04% (39.693- 71.19).

**Conclusion:**

A single dose of nirsevimab given before or during the RSV season demonstrated a significant and sustained impact on RSV LRTI hospitalisations for the entire RSV season. Consistent efficacy was shown across subgroups comprising the all infant population.The potential impact of nirsevimab was reinforced by a reduction in all cause LRTI hospitalisations.

**Disclosures:**

**Saul N. Faust, FRCPCH PhD**, AstraZeneca, Janssen, Pfizer, Moderna, GlaxoSmithKline, Novavax, Sanofi, Seqirus, Medimmune, Merck, MSD, Iliad and Valneva: Advisor/Consultant|AstraZeneca, Janssen, Pfizer, Moderna, GlaxoSmithKline, Novavax, Sanofi, Seqirus, Medimmune, Merck, MSD, Iliad and Valneva: Investigator **Katrina Cathie, MBE, FRCPCH**, AstraZeneca: Advisor/Consultant|GSK: Advisor/Consultant|Iliad: Advisor/Consultant|Janssen: Advisor/Consultant|MedImmune: Advisor/Consultant|Merck: Advisor/Consultant|Pfizer: Advisor/Consultant|Sanofi: Advisor/Consultant|Valneva: Advisor/Consultant **SB Drysdale, FRCPCH, PhD**, AstraZeneca: Advisor/Consultant|iLiAD: Advisor/Consultant|Janssen: Advisor/Consultant|Moderna: Advisor/Consultant|MSD: Advisor/Consultant|Pfizer: Advisor/Consultant|Sanofi: Advisor/Consultant|Valneva: Advisor/Consultant **S Royal, FRCGP**, Sanofi: Advisor/Consultant **C Felter, MD**, Sanofi: Employee **NC Vassilouthis, MD**, Sanofi: Employee **Mathieu Bangert, PhD**, Sanofi: Staff member **K Mari, PhD**, Sanofi: Employee **R Nteene, MD**, Sanofi: Employee **M Roberts, MD**, Sanofi: Employee **P Tissieres, MD**, Baxter: Advisor/Consultant|PAion: Advisor/Consultant|Sanofi: Advisor/Consultant|Sedana: Advisor/Consultant

